# Predictive value of serum glutathione S-transferase (GST-â), P-glycoprotein (PGP), P53, KI-67 in breast cancer: A systematic review and meta-analysis

**DOI:** 10.5937/jomb0-56147

**Published:** 2025-08-21

**Authors:** Nuan Zhang, Zhipeng Wang, Yang Zhang

**Affiliations:** 1 Shandong University of Traditional Chinese Medicine Affiliated Hospital, Department of Oncology, Jinan, Shandong, China; 2 Shandong University of Traditional Chinese Medicine Affiliated Hospital, Department of Pulmonary and Critical Care Medicine, Jinan, Shandong, China

**Keywords:** serum glutathione S-transferase (GST-â), P-glycoprotein (PGP), P53, Ki-67, triple-negative breast cancer, chemotherapy resistance, drug-resistant protein, meta-analysis, serumska glutation-S-transferaza (GST-â), P-glikoprotein (PGP), P53, Ki-67, trostruko negativni karcinom dojke, rezistencija na hemioterapiju, proteini rezistentni na lekove, meta-analiza

## Abstract

**Background:**

To assess the predictive value of drug-resistant proteins - serum glutathione S-transferase (GST-â), P-glycoprotein (PGP), P53, Ki-67 - in triple-negative breast cancer (TNBC) and their role in chemotherapy resistance. This systematic review and meta-analysis aimed to explore their clinical relevance for improving TNBC treatment outcomes.

**Methods:**

We systematically searched PubMed, Web of Science, CNKI, WanFang, and VIP databases for studies from 2010 to 2024. Studies meeting predefined inclusion and exclusion criteria were selected. Data extraction and quality assessment were performed by two independent researchers. Meta-analysis was conducted using RevMan 5.3 software.

**Results:**

Seven studies were included, involving 1,772 patients, with 745 TNBC cases and 1,027 non-TNBC cases. Meta-analysis showed that in TNBC compared to non-TNBC, the expression rates of GST-â [O R= 3.41, 95% CI (2.21, 5.25), P< 0.00001], PGP [O R= 1.87, 95% CI (1.17, 2.98), P= 0.008], P53 [O R= 3.65, 95% CI (2.25, 5.91), P< 0.00001], and Ki-67 [O R= 1.19, 95% CI (0.54, 1.84), P= 0.0004] were significantly elevated, indicating higher drug resistance. However, no significant differences were found in Topo I, II, or III expression. Additionally, TNBC patients had poorer disease-free survival (DFS) [O R = 0.30, 95% CI (0.15, 0.59), P=0.0005] and overall survival (OS) [O R=0.17, 95% CI (0.11, 0.28), P<0.00001] compared to non-TNBC patients.

**Conclusions:**

The elevated expression of drug-resistant proteins GST-â, PGP P53, and Ki-67 in TNBC suggests that these biomarkers are closely associated with chemotherapy resistance. Monitoring their levels during treatment may help guide more effective clinical strategies for managing TNBC. The findings emphasise the need for personalised therapeutic approaches based on protein expression profiles to improve clinical outcomes for TNBC patients.

## Abstract (sr)

**Uvod:**

Procena prediktivne vrednosti proteina povezanih sa rezistencijom na lekove - serumske glutation-S-transferaze (GST-â), P-glikoproteina (PGP), P53 i Ki-67 - kod trostruko negativnog karcinoma dojke (TNBC) i njihove uloge u rezistenciji na hemioterapiju. Ova sistematska analiza i meta-analiza imale su za cilj da istraže njihovu kliničku relevantnost u poboljšanju ishoda lečenja TNBC-a.

**Metode:**

Sistematski su pretražene baze podataka PubMed, Web of Science, CNKI, WanFang i VIP za studije objavljene u periodu od 2010. do 2024. godine. Odabrane su studije koje su ispunjavale unapred definisane kriterijume uključivanja i isključivanja. Ekstrakciju podataka i procenu kvaliteta studija sproveli su dva nezavisna istraživača. Meta-analiza je izvedena korišćenjem softvera RevMan 5.3.

**Rezultati:**

Uključeno je sedam studija sa ukupno 1.772 pacijentkinje, od kojih je 745 imalo TNBC, a 1.027 druge tipove karcinoma dojke. Meta-analiza je pokazala da su kod TNBC-a, u poređenju sa drugim tipovima, stope ekspresije GST-â [O R=3,41, 95% CI (2,21, 5,25), P<0,00001], PGP [OR= 1,87, 95% CI (1,17, 2,98), P=0,008], P53 [O R=3,65, 95% CI (2,25, 5,91), P<0,00001] i Ki-67 [O R=1,19, 95% CI (0,54, 1,84), P=0,0004] bile značajno povišene, što ukazuje na viši nivo rezistencije na lekove. Međutim, nije pronađena značajna razlika u ekspresiji Topo I, II ili III. Pored toga, pacijentkinje sa TNBC-om su imale lošiji ishod u pogledu preživljavanja bez bolesti (DFS) [O R=0,30, 95% CI (0,15, 0,59), P=0,0005] i ukupnog preživljavanja (OS) [O R=0,17, 95% CI (0,11, 0,28), P<0,00001] u odnosu na pacijentkinje sa drugim vrstama karcinoma dojke.

**Zaključak:**

Povišena ekspresija proteina rezistentnih na lekove GST-â, PGP P53 i Ki-67 kod TNBC-a ukazuje na njihovu usku povezanost sa rezistencijom na hemioterapiju. Praćenje nivoa ovih biomarkera tokom terapije može pomoći u usmeravanju efikasnijih kliničkih strategija za lečenje TNBC-a. Nalazi naglašavaju potrebu za personalizovanim terapijskim pristupima zasnovanim na ekspresionim profilima proteina kako bi se poboljšali klinički ishodi kod pacijentkinja sa TNBC-om.

## Introduction

Breast cancer remains one of the most prevalent and challenging health conditions affecting women globally, with an alarmingly high incidence rate that significantly impacts both the physical and mental well-being of women. As one of the leading causes of cancer-related deaths in women, breast cancer accounts for a substantial proportion of global mortality rates in this demographic [1] [2] [3]. Within the broad spectrum of breast cancer, there exists a particularly aggressive and difficult-to-treat subtype known as Triple-Negative Breast Cancer (TNBC). This subtype is defined by the absence of three key receptors: estrogen receptor (ER), progesterone receptor (PR), and human epidermal growth factor receptor 2 (HER2) [4] [5]. These receptors, which play a crucial role in the growth and proliferation of many breast cancer cells, are typically targeted in standard breast cancer therapies. However, TNBC cells lack these receptors, making traditional therapeutic strategies such as hormone therapy and HER2-targeted treatments ineffective [6] [7].

Despite constituting only 10%-20% of all breast cancer diagnoses [8], TNBC is associated with a significantly poorer prognosis compared to other types of breast cancer. Patients diagnosed with TNBC often face more aggressive tumour growth, higher rates of metastasis, and poorer overall survival rates [9]. This poor prognosis is compounded by the fact that TNBC tends to occur in younger women and is more common among women of African or Hispanic descent, further emphasising the need for targeted research and improved treatment options for this population. Treatment strategies for TNBC typically involve chemotherapy, often in combination with targeted therapies; however, these approaches are not always effective [10].

One of the significant challenges in treating TNBC is its high level of resistance to multiple drugs, a phenomenon known as multidrug resistance (MDR). This resistance is primarily driven by the abnormal expression of multidrug resistance proteins, such as ATP-binding cassette (ABC) transporters, which pump chemotherapeutic agents out of cancer cells before they can exert their therapeutic effects. As a result, TNBC patients often experience limited success with chemotherapy, leading to the need for alternative or adjunctive therapeutic strategies. Additionally, the molecular mechanisms behind developing multidrug resistance in TNBC are not yet fully understood, presenting a significant barrier to developing effective treatments.

To better understand the complexities of TNBC and the reasons behind its aggressive nature and poor response to treatment, this paper aims to analyse and compare the expression of relevant proteins in TNBC versus non-TNBC cases. Through meta-analysis, this study seeks to provide a more comprehensive picture of the molecular underpinnings of TNBC, particularly about the expression of multidrug resistance proteins and other key biomarkers. By examining existing literature and aggregating data from various studies, this analysis will help clarify the role of these proteins in the progression of TNBC and potentially reveal novel therapeutic targets that could improve treatment outcomes for TNBC patients. Ultimately, the goal is to contribute to the development of more effective, personalised treatment strategies that can overcome the challenges posed by this difficult-to-treat cancer subtype.

## Materials and methods

### Document inclusion criteria

In this meta-analysis, we established several inclusion criteria to ensure the relevance and comprehensiveness of the studies included. First, all studies must focus exclusively on patients diagnosed with Triple-Negative Breast Cancer (TNBC), excluding those involving other breast cancer subtypes or mixed populations. The studies had to be published in English to standardise the language and facilitate effective data extraction. Only case-control studies were considered, as they provide a solid basis for comparing TNBC patients with control groups regarding biomarker expression and clinical outcomes. Furthermore, studies with accessible full texts were included to ensure adequate data could be extracted for analysis. We also excluded studies involving patients with multiple cancers, ensuring that the research addressed TNBC.

### Document exclusion criteria

We applied several exclusion criteria to maintain the rigour of the analysis. Studies published in languages other than English were excluded to avoid complications with translation and to standardise the dataset. Any study design other than a case-control study, such as cohort studies, cross-sectional studies, or randomised controlled trials, was excluded, as they do not offer the same comparative framework relevant to our analysis. We also excluded studies where the outcome indicators were unclear or incomplete, particularly concerning protein expression levels like GST-π, Pgp, P53, Ki-67, and Topo, or clinical outcomes such as Disease-Free Survival (DFS) and Overall Survival (OS). Conference papers and review articles were excluded due to their often limited methodological detail, which is necessary for robust meta-analysis. Studies where the full text was inaccessible or unavailable were also excluded to ensure we could extract sufficient data for the analysis. Lastly, we removed duplicate publications to prevent redundancy and potential bias in the data.

### Literature search strategy

We conducted a comprehensive literature search across multiple databases to ensure we included all relevant studies. These databases included PubMed, Web of Science, CNKI (China National Knowledge Infrastructure), WanFang Data, and VIP (Chinese Scientific Journals Database), focusing on publications from 2010 to 2024.

WanFang Data is a major Chinese database that provides access to many scholarly resources, including journals, dissertations, and conference proceedings, particularly in health sciences and medicine. It is valuable for accessing Chinese-language studies that may not be available in international databases like PubMed or Web of Science. Including WanFang in your literature search ensures a more comprehensive collection of studies, particularly those relevant to Chinese-speaking populations.

We used a combination of keywords, such as »Triple Negative Breast Cancer,« »TNBC,« »GST-π,« »Pgp,« »P53,« »Ki-67,« and »Topo.« We combined them using Boolean operators (AND, OR) to capture all studies examining the expression of these biomarkers in TNBC and their correlation with clinical outcomes like DFS and OS. The goal was to include studies that reported on the protein expression levels of interest and their potential impact on the prognosis of TNBC patients.

### Literature extraction and quality evaluation

Two researchers independently screened the relevant studies and extracted the necessary data according to the established inclusion and exclusion criteria. This data included details such as the author and publication year, study design, sample size, and biomarker expression levels for GST-π, P-glycoprotein (Pgp), P53, Ki-67, and Topoisomerase (Topo) grades I, II, and III, as well as clinical outcomes (DFS and OS). If any disagreements arose between the two researchers during data extraction, a third researcher was consulted to resolve the issue. This ensured the accuracy and consistency of the data collected. The quality of the included studies was assessed using the Newcastle-Ottawa Scale (NOS), which evaluates studies based on selection, comparability, and outcome. Studies that scored less than 6 out of 10 on the NOS were considered poor quality and excluded from the meta-analysis.

### Statistical analysis

Meta-analysis was performed using RevMan 5.3 software, a widely used tool for conducting systematic reviews and meta-analyses in the biomedical field. We used odds ratios (OR) and 95% confidence intervals (CI) as the primary effect size measures to quantify the associations between protein expression levels and clinical outcomes in TNBC patients. Heterogeneity among the included studies was assessed using the P-value and I^2^ statistic. If no significant heterogeneity was detected (P>0.1 and I^2^< 50%), a fixed-effect model was applied to combine the data. A random-effects model was used instead if significant heterogeneity was found (P<0.1 and I^2^ >50%). To account for potential sources of heterogeneity, subgroup analysis was conducted when appropriate, examining variations in biomarker expression across different patient populations. A sensitivity analysis was also performed to assess the robustness of the results by excluding individual studies and observing how this impacted the overall effect estimates. The final output of the meta-analysis included point estimates and 95% CI for the association between biomarker expression and clinical outcomes such as DFS and OS in TNBC patients.

## Results

### Basic features of the included literature

**Figure 1:**
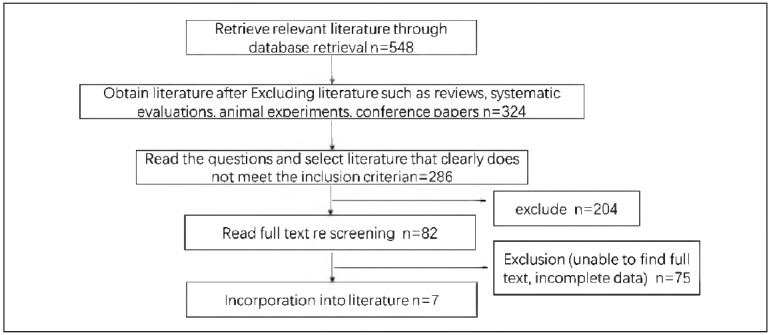
Literature screening process.

Based on the inclusion and exclusion criteria, seven articles [11] [12] [13] [14] [15] [16] [17] were contained, all in English, with a total number of 1772 patients. Among them, 745 cases of TNBC were contained in the study group (SG), and 1027 cases of non-TNBC were contained in the control group (CG). The NOS scores of the included literature were all higher than 5, reflecting that the included literature was of high quality and could be used for meta-analysis. The literature screening process and basic characteristics are displayed in Figure 1 and Table 1.

**Table 1:** Basic features of the included literature. ①GST-π ②Pgp ③P53 ④Ki-67 ⑤Topo grade ⑥Topo grade ⑦Topo grade ⑧DFS ⑨OS

Included literature	Year of publication	NOS score	Outcome indicator	Number of patients/cases	
				Study group	Control group
Wang Li [11]	2014	7	①②③④⑤⑥⑦	33	60
Wei Wang [12]	2016	8	④⑤⑥⑦⑧⑨	167	196
Ling Zhou MD [13]	2013	9	②④⑤⑥⑦	31	88
Bhumsuk Keam [14]	2011	9	③④⑧⑨	105	105
Liang Huang [15]	2013	8	④⑧⑨	319	185
Jae Jeong Choi [16]	2010	8	②③④	43	188
SANG Jing [17]	2011	8	⑥	47	205

### Meta-analysis results

### GST-π

**Figure 2:**
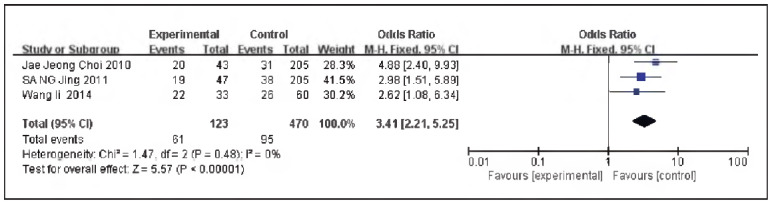
Forest map of GST-π expression in TNBC and non-TNBC.

3 documents [11] [16] [17] were included, including 123 cases in the SG and 470 cases in the CG. No heterogeneity could be discovered among the studies (P=0.48, I^2^ = 0%), so the fixed effect model was implemented for meta-analysis. The outcomes revealed the expression of GST-π protein in the SG and the CG was significant [OR=3.41, 95% CI (2.21, 5.25), P<0.00001], and the expression rate of GST-π protein in TNBC was elevated in contrast to non-TNBC (Figure 2).

### Pgp

**Figure 3:**
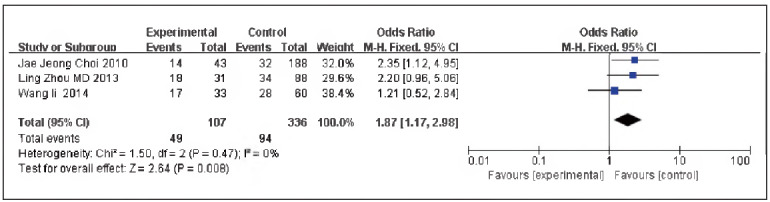
Forest map of Pgp expression in TNBC and non-TNBC.

A total of 3 pieces of literature were contained [11] [13] [16], including 107 cases in the SG and 336 cases in the CG. No heterogeneity could be discovered among the studies (P=0.47, I^2^=0%), so the fixed-effect model was implemented for meta-analysis. The outcomes unveiled that the expression of Pgp protein in the SG and the CG was significant [OR=1.87, 95% CI (1.17, 2.98), P=0.008], and the expression rate of Pgp protein in TNBC was elevated in contrast to non-TNBC (Figure 3).

### P53

**Figure 4:**
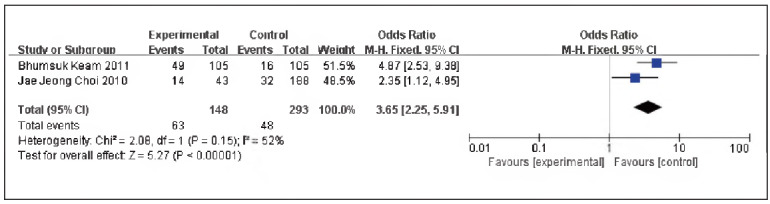
Forest map of P53 expression TNBC and non-TNBC.

A total of 2 pieces of literature were contained [14] [16], including 148 cases in the SG and 293 cases in the CG. No heterogeneity could be discovered among the studies (P=0.15, I^2^ = 52%), so the fixed-effect model was implemented for meta-analysis. The outcomes displayed the expression of P53 protein in the SG and the CG had significance [OR=3.65, 95% CI (2.25, 5.91), P<0.00001], and the expression rate of P53 protein in TNBC was elevated in contrast to non-TNBC (Figure 4).

### Ki-67

**Figure 5:**
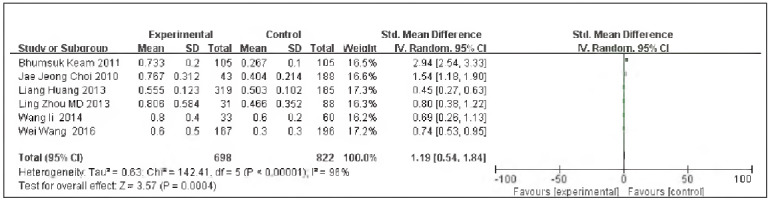
Forest map of Ki-67 expression in TNBC and non-TNBC.

Six papers [11] [12] [13] [14] [15] [16] were included, including 698 cases in the SG and 822 cases in the CG. There was heterogeneity among the studies (P<0.00001, I^2^ = 96%). Therefore, the random effects model was implemented for meta-analysis, and the outcomes showed the expression of Ki-67 in the SG and the CG was significant [OR=1.19, 95% CI (0.54, 1.84), P=0.0004], and the expression rate of Ki-67 in TNBC was elevated in contrast to non-TNBC (Figure 5).

### Topo grade I level

**Figure 6:**
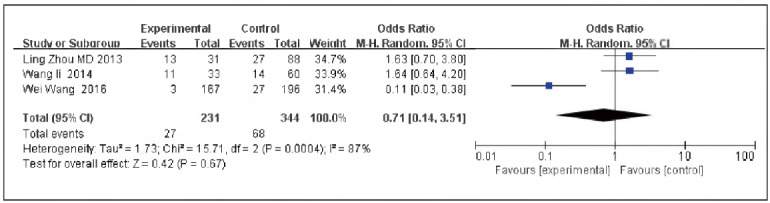
Forest map of Topo grade I in TNBC and non-TNBC.

A total of 3 papers [11] [12] [13] were contained, including 231 cases in the SG and 344 cases in the CG. There was heterogeneity among the studies (P=0.0004, I^2^ =87%). Therefore, the random effects model was implemented for meta-analysis. The outcomes manifested the expression of Topo grade in the SG, and the CG was not significant [OR=0.71, 95% CI (0.14, 3.51), P=0.67], and the expression rate of Topo grade in TNBC was elevated in contrast to non-TNBC (Figure 6).

### Topo grade II

**Figure 7:**
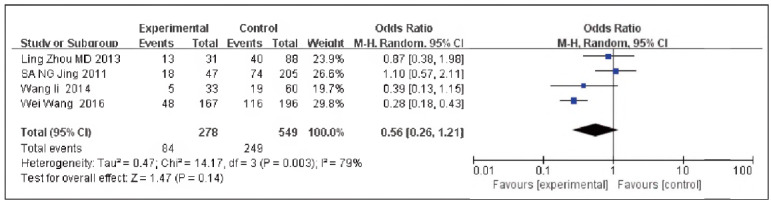
Forest map of Topo grade II in triple-negative and non-TNBC.

A total of 4 papers [11] [12] [13] [17] were contained, including 278 cases in the SG and 549 cases in the CG. There was heterogeneity among the studies (P=0.003, I^2^ = 79%). Therefore, the random effects model was implemented for meta-analysis. The outcomes displayed the expression of Topo grade in the SG, and the CG was not significant [OR=0.56, 95% CI (0.26, 1.21), P=0.14], and the expression rate of Topo grade in TNBC was elevated in contrast to non-TNBC (Figure 7).

### Topo grade III

**Figure 8:**
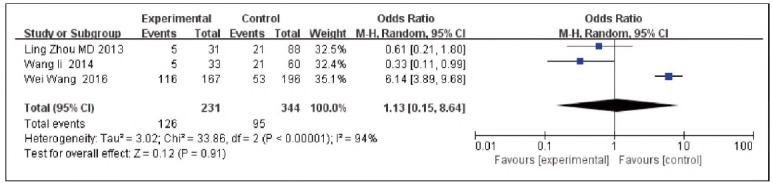
Forest map of Topo grade III in TNBC and non-TNBC.

A total of 3 papers [11] [12] [13] were contained, including 231 cases in the SG and 344 cases in the CG. There was heterogeneity among the studies (P<0.00001, I^2^ = 94%). Therefore, the random effects model was implemented for meta-analysis. The outcomes presented the expression of Topo grade in the SG, and the CG was not significant [OR=1.13, 95% CI (0.15, 8.64), P=0.91], and the expression rate of Topo grade in TNBC was elevated in contrast to non-TNBC (Figure 8).

### DFS

**Figure 9:**
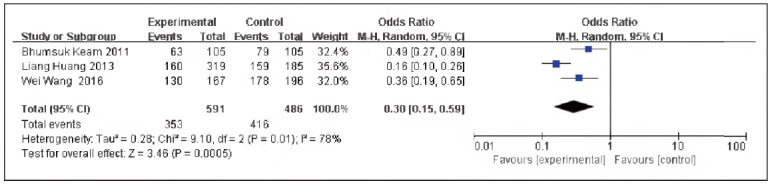
Forest map of DFS level in TNBC and non-TNBC.

A total of 3 papers [12] [14] [15] were included, including 591 cases in the SG and 486 cases in the CG. There was heterogeneity among the studies (P=0.01, I^2^ = 78%). Therefore, the random effects model was implemented for meta-analysis. The outcomes exhibited that the DFS levels in the SG and the CG were significant [OR=0.30, 95% CI (0.15, 0.59), P=0.0005], and the DFS levels in TNBC were elevated in contrast to non-TNBC (Figure 9).

### OS

**Figure 10:**
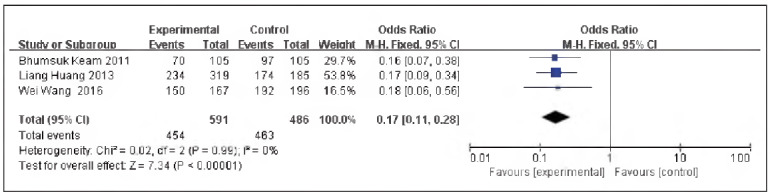
Forest map of OS level in TNBC and non-TNBC.

A total of 3 literatures were included [12] [14] [15], including 591 cases in the SG and 486 cases in the CG. No heterogeneity could be discovered among the studies (P=0.99, I^2^ = 0%). Therefore, the fixed effect model was implemented for meta-analysis, and the outcomes revealed the levels of OS in the SG and the CG were significant [OR=0.17, 95% CI (0.11, 0.28), P<0.00001], and the levels of OS in TNBC were elevated in contrast to non-TNBC (Figure 10).

## Discussion

As a common case reaction of breast cancer, TNBC can be evaluated by three biological markers, ER, PR and Her-2, to propose specific therapy for breast cancer. With ER and Her-2 as targets, patients with receptor-positive TNBC can be clinically treated [18]. However, due to the significant gene polymorphism of breast cancer patients, their pathological biomarkers are significantly different. TNBC is highly invasive and challenging to adapt to conventional treatment. Therefore, TNBC patients have a poor prognosis [19]. Common targeted therapies are not suitable for the treatment of TNBC. Chemotherapy is a common treatment for TNBC, but it is still only effective for a small number of patients [20]. Therefore, it is necessary to analyse the mechanism of multidrug resistance in TNBC.

GST-π is one of the main members of the GSTs family, and the main mechanism is to degrade drugs by catalysing glutathione and various electrophilic compounds, which weakens the therapeutic effect of chemotherapy drugs on tumours and thus enhances the drug resistance of cancer cells. Therefore, in general, GST-π expression in breast cancer tissues is very high, often much higher than that in non-breast cancer tissues, and the prognosis for patients is poor. Therefore, some researchers have pointed out that a targeted chemotherapy regimen can be adopted by observing the expression of GST-π in patients with TNBC, which can effectively improve the efficacy of chemotherapy [21]. The outcomes of this study revealed that the expression level of GST-π was increased in TNBC in contrast to non-TNBC, P<0.00001, indicating that the drug resistance of TNBC is relatively strong. The high level of GST-π expression may be one of the important factors of drug resistance, which can provide a basis for clinical screening of effective chemotherapy drugs.

Pgp is a drug discharge pump encoded by multidrug resistance genes, which pumps anti-tumour drugs from intracellular to extracellular, and the intracellular concentration of drugs keeps decreasing, thus enabling cells to obtain drug resistance. This mechanism has been confirmed in clinical practice [22]. It can be expressed in almost all tumour cells, and the expression level of tumours that are insensitive to chemotherapy or have poor efficacy is often higher. The outcomes of this study also displayed that the expression rate of Pgp protein in TNBC was elevated in contrast to non-TNBC, P=0.008.

The relationship between P53 protein and TNBC has always been a research hotspot, and the relationship between the two has always been controversial. Still, the high expression of P53 protein in TNBC is recognised, and the lymphatic metastasis rate and OS of patients with TNBC may be affected. This study's outcomes also showed that the expression rate of P53 protein in TNBC was elevated compared to non-TNBC (P<0.00001). Therefore, the expression of P53 protein should also be considered in treating TNBC.

Ki-67 is a large molecular nuclear protein localised in the nucleus and associated with proliferation. It is expressed in all cell proliferation cycles except the G-0 phase [14]. The outcomes of this study presented that the expression rate of Ki-67 in TNBC was elevated in contrast to non-TNBC (P=0.006), the DFS level in TNBC was elevated in comparison to non-TNBC (P=0.0005), and the OS level in TNBC was elevated in contrast to non-TNBC. P<0.00001, which was fully consistent with the biological characteristics of rapid growth and intense aggressiveness of TNBC, indicating that Ki-67 has a specific correlation with drug resistance of TNBC, but its specific mechanism needs to be further studied.

Topo grade II is a key enzyme that acts on nucleic acids and binds to DNA to form a momentary gap in the DNA double-strand of replication. If Topo II is inhibited or cleaved, it will lead to a permanent gap in the DNA double-strand and cell death. As the target of anthracyclines, changes in Topo II may affect treatment sensitivity, which is related to drug resistance such as doxorubicin and daunorubicin [23]. However, the outcomes of this study displayed no difference in the expression rate of Topo grade II between TNBC and non-TNBC, which may be related to the low sample size contained in this study.

Faur and colleagues [24] explored the predictive role of serum lipid levels, p53, and Ki-67 in breast cancer patients undergoing neoadjuvant chemotherapy (NAC), with a focus on the molecular subtypes of the disease. They identified key factors, such as tumour size and genetic markers like Ki-67 and p53, that correlated with overall survival (OS) and disease- free survival (DFS), with distinct lipid profile changes influencing treatment response, particularly in triplenegative breast cancer (TNBC). In contrast, our study focuses on drug-resistant proteins - GST-π, P-glycoprotein, p53, and Ki-67 - in chemotherapy resistance, specifically in TNBC. We found that elevated levels of these proteins were associated with poorer survival outcomes and increased resistance to chemotherapy in TNBC patients, suggesting their importance as biomarkers for treatment strategies. While both studies highlight the predictive value of Ki-67 and p53, our meta-analysis emphasises the clinical significance of drug-resistant proteins in TNBC therapy, whereas Faur and colleagues focus more on lipid profiles and tumour characteristics across various breast cancer subtypes.

In another study, Song and colleagues [25] examined the combined diagnostic and prognostic value of HER2, Ki67, and GSTP1 in breast cancer, finding correlations with tumour size, lymph node metastasis, and TNM stage. Both studies highlight the importance of Ki-67 and GSTP1, but while Song et al. focus on gene expression and méthylation for diagnosis, our study explores protein markers related to treatment resistance.

In a recent study, Finkelman and colleagues [26] reviewed the evolution of Ki-67 as a biomarker for breast cancer, highlighting its importance in assessing cellular proliferation, prognosis, and chemotherapy response. While Ki-67 is widely recognised, they emphasised the need for standardisation in its assay and interpretation to improve its prognostic and predictive value. Both studies recognise Ki-67 as a crucial biomarker, but Finkelman et al. focused on its historical development and clinical application, while our study investigates its role in chemotherapy resistance.

In another study by Marvalim and colleagues [27], they reviewed the role of p53 in breast cancer progression, emphasising its regulatory functions in response to genotoxic stress and its frequent inactivation through mutation or MDM2 overexpression. They highlighted the subtype-specific nature of p53 mutations and discussed emerging therapies targeting both wild-type and mutant p53, such as small molecules, peptides, and PROTACs. Our study also explores p53 as a biomarker concerning chemotherapy resistance in TNBC. Both studies underline the importance of p53 in breast cancer. Still, while Marvalim et al. focus on therapeutic strategies to target p53 in various subtypes, our study investigates its role in predicting treatment response, particularly in TNBC.

Limitations of this study: (1) The included literature only limited the English literature from 2010 to 2024, and all of them contained the outcome indicators of this study, so there were fewer included papers and a small sample size, which may affect the conclusion of this study. Future studies need to increase the literature to verify the research conclusions further. (2) Although the quality of the contained literature is relatively high, this study did not analyse the sensitivity of the research, which may affect the conclusion of the article. (3) Since meta-analysis is based on the original study, it will be affected by the original literature. Hence, future studies need to improve the quality of the included literature to better guide clinical practice.

## Conclusion

This meta-analysis highlights the elevated expression of drug-resistant proteins (GST-π, PGP, P53, and Ki-67) in triple-negative breast cancer (TNBC), suggesting a strong link to chemotherapy resistance and poor clinical outcomes. Monitoring these biomarkers could help guide more personalised treatment strategies, improving the management and prognosis of TNBC. These findings underscore the importance of incorporating protein expression profiles into clinical practice to optimise therapy and overcome resistance in TNBC patients. Further research with larger sample sizes is needed to confirm these results and refine treatment approaches.

## Dodatak

### Acknowledgements

The authors thank the researchers whose studies were included in this meta-analysis. Their contributions were invaluable to the success of this research. We also appreciate the support provided by the Department of Clinical Oncology at the Affiliated Hospital of Shandong University of TCM.

### Funding

The Science and Technology Project of Traditional Chinese Medicine in Shandong (No. 2021Q109) supported this study.

### Authors' contribution

Nuan Zhang: Conceptualisation, methodology, data collection, data analysis, writing the original draft.

Zhipeng Wang: Conceptualisation, supervision, writing, and reviewing the manuscript.

Yang Zhang: Data collection, data analysis, manuscript editing.

### Conflict of interest statement

All the authors declare that they have no conflict of interest in this work.
